# Supplementary material for: Asymmetric Synthesis of Quaternary Hydantoins via a Palladium-Catalyzed Aza-Heck Cyclization
Source: J Am Chem Soc. 2025 Nov 14;147(49):44692–8. doi: 10.1021/jacs.5c16022 (PMC12703750; doi:10.1021/jacs.5c16022)
Supplement: Supplementary file 2 [file ja5c16022_si_002.zip › All NMR FID Files/S17/S17_AllNMR/14/pdata/1/email_TDI01061-xterize again2_14_1.pdf]

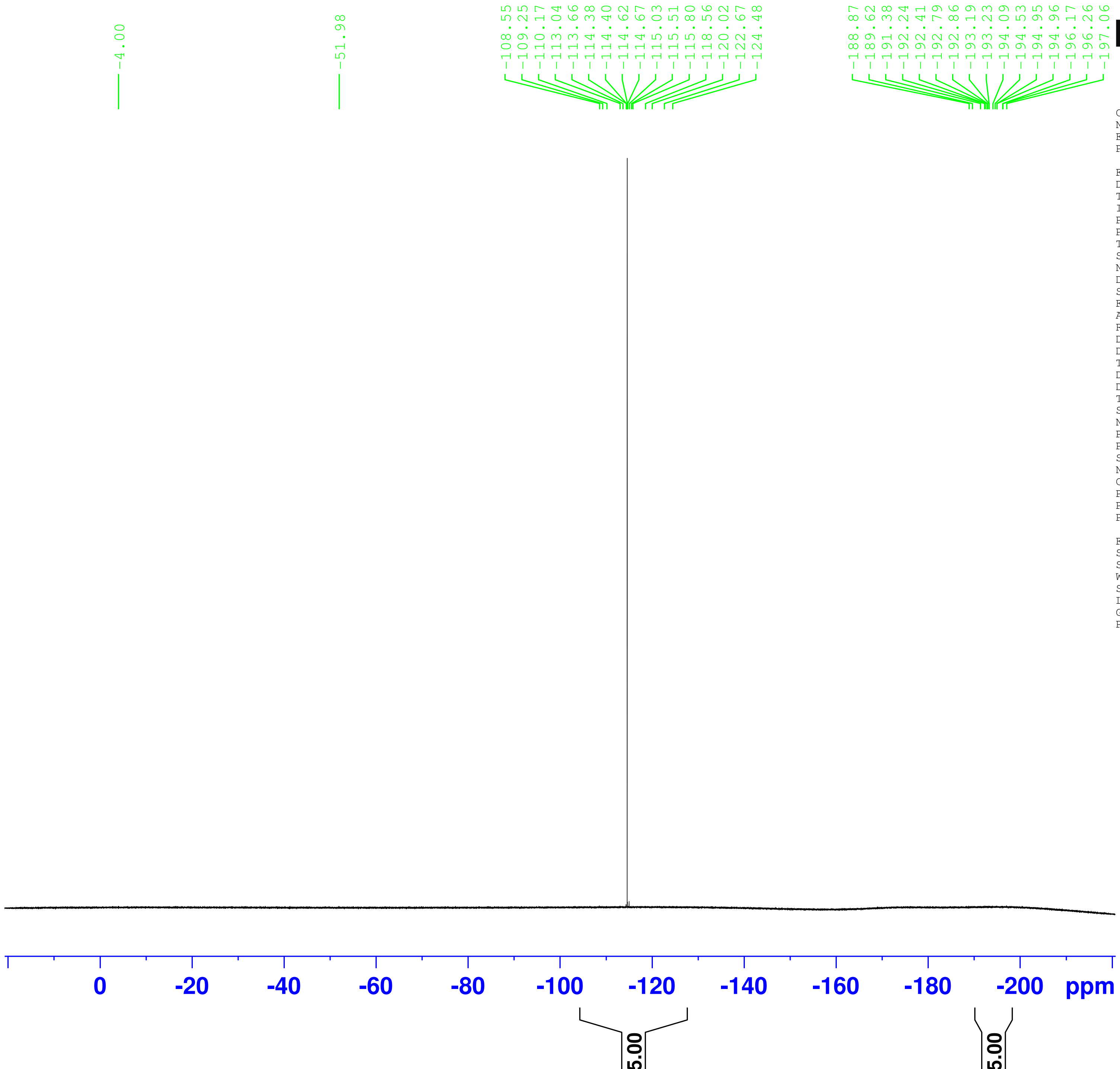

Current Data Parameters

NAME	TDI01061-xterize again2	
EXPNO	14	
PROCNO	1	

F2 - Acquisition Parameters

Date_	20250109	
Time	7.40	h
INSTRUM	Avance Neo 400	U of DE Brown Lab
PROBHD	Z104450_0412 (	
PULPROG	zgig	
TD	131072	
SOLVENT	CDCl3	
NS	32	
DS	4	
SWH	90909.091	Hz
FIDRES	1.387163	Hz
AQ	0.7208960	sec
RG	101	
DW	5.500	usec
DE	6.50	usec
TE	298.0	K
D1	1.00000000	sec
D11	0.03000000	sec
TD0	1	
SFO1	376.4607164	MHz
NUC1	19F	
P1	15.00	usec
PLW1	11.81599998	W
SFO2	400.1316005	MHz
NUC2	1H	
CPDPRG[2	waltz16	
PCPD2	90.00	usec
PLW2	8.31639957	W
PLW12	0.23101000	W

F2 - Processing parameters

SI	65536	
SF	376.4983662	MHz
WDW	EM	
SSB	0	
LB	0.30	Hz
GB	0	
PC	1.00	
